# Effects of Green Light Deprivation and Red-to-Blue Ratio on Growth, Mineral Content, and Pigments in *Salvia officinalis* L. and *Cannabis sativa* L.

**DOI:** 10.3390/plants15071004

**Published:** 2026-03-25

**Authors:** Shaimaa Mousa Mohamed Hussein, Massimiliano D’Imperio, Vittorio Napolitano, Giuseppe di Cuia, Angela Boari, Angelo Parente, Francesco Serio

**Affiliations:** Institute of Sciences of Food Production, National Research Council of Italy (CNR), Via G. Amendola, 122/O, 70126 Bari, Italy; shaimaamousamohamedhussein@cnr.it (S.M.M.H.); vittorionapolitano@cnr.it (V.N.); giuseppedicuia@cnr.it (G.d.C.); angela.boari@cnr.it (A.B.); angelo.parente@cnr.it (A.P.); francesco.serio@cnr.it (F.S.)

**Keywords:** LED lighting, light spectrum, photosynthetic pigments, mineral composition, controlled environment agriculture, medicinal plants

## Abstract

Light spectral composition plays a central role in regulating plant growth, morphology, nutrient uptake, and pigment biosynthesis, particularly in controlled-environment agriculture. This study investigated the effects of targeted LED spectral modulation, focusing on green light deprivation and different red-to-blue (R:B) ratios at constant photon flux density, on morphological traits, mineral composition, and photosynthetic pigments in *Salvia officinalis* L. and *Cannabis sativa* L. grown under controlled conditions. Plants were cultivated under three LED treatments providing equal light intensity but differing in spectral composition. Morphological parameters, mineral nutrients, inorganic anions, and photosynthetic pigments were assessed at harvest. Total biomass production was not significantly affected by the light treatments in either species; however, clear species-specific responses were observed. In *S. officinalis*, higher R:B ratios promoted stem elongation without affecting leaf number or fresh weight, whereas in *C. sativa*, the higher R:B ratio significantly increased leaf number. Green light deprivation and red–blue enrichment generally enhanced mineral accumulation and nitrogen content, although the magnitude and direction of these effects varied between species. Photosynthetic pigment responses were more pronounced in hemp, with increased chlorophylls and carotenoids under green light deprivation, while salvia showed a selective increase in carotenoids under higher R:B ratios. Overall, these findings emphasize the importance of species-specific LED spectral optimization to improve physiological performance and nutritional quality in indoor cultivation of medicinal plants.

## 1. Introduction

Light represents not only the main source of energy driving photosynthesis but also a critical environmental signal that orchestrates plant growth, morphogenesis, and metabolism. Its spectral composition influences multiple physiological and biochemical processes, affecting plant structure, photosynthetic efficiency, and secondary metabolism. The ability to manipulate light quality, intensity, and duration has therefore become a central strategy for improving crop performance and quality in indoor systems [1,2,3]. In recent years, the rapid development of light-emitting diode (LED) technology has revolutionized horticultural lighting, allowing researchers and growers to finely adjust the spectral output according to species-specific requirements [4].

This flexibility enables precise spectral control and the creation of light environments tailored to stimulate targeted physiological responses. Within the photosynthetically active radiation (PAR) range (400–700 nm), each spectral region plays distinct roles in plant biology. Blue light (400–500 nm) is primarily associated with photomorphogenesis, chlorophyll synthesis, and the regulation of stomatal conductance, as it activates specific photoreceptors such as cryptochromes and phototropins. Red light (600–700 nm) is crucial for photosystem activation, phytochrome-mediated signaling, and the regulation of flowering and elongation processes. In contrast, green light (500–600 nm), once considered less relevant for photosynthesis, has been shown to penetrate deeper into the canopy, contributing to photosynthetic activity in shaded tissues and improving the overall light-use efficiency of plants [5,6,7,8,9,10,11].

Although the physiological role of green light in plants is not yet fully understood, the nature of the green-light receptor remains controversial. Several studies suggest that green light may be perceived indirectly through cryptochromes (CRYs), which are primarily blue-light photoreceptors [6,7]. In this context, green light has been proposed to partially counteract blue-light responses through an antagonistic blue–green interaction mediated by the interconversion of flavin redox states within cryptochromes. This mechanism may modulate plant photomorphogenic responses and influence physiological processes regulated by blue light [6,7].

Taking into account both this interaction and the significant contribution of green wavelengths to the photosynthetically active radiation (PAR) spectrum, the removal of wavelengths between 500 and 600 nm may likewise impact plant growth, productivity, and key physiological processes.

The interaction between these spectral regions defines plant morphology and metabolism. In particular, the red-to-blue light ratio has profound implications for plant architecture, chlorophyll accumulation, and photosynthetic capacity. Higher proportions of blue light typically result in shorter, more compact plants with increased chlorophyll and secondary metabolite content, while red-enriched spectra stimulate elongation and biomass production, albeit sometimes at the expense of leaf thickness or pigment concentration [12,13]. Recent studies have highlighted the importance of adding green or far-red wavelengths to improve canopy photosynthesis and regulate shade-avoidance responses, emphasizing the need for species-specific optimization of light recipes [14,15]. Beyond morphological and photosynthetic responses, light quality also modulates mineral uptake and nutrient partitioning. Variations in spectral composition can influence ion transport, root morphology, and the accumulation of macro- and micronutrients such as nitrogen, potassium, calcium, and magnesium [1]. Red–blue combinations often enhance nutrient assimilation and pigment biosynthesis, while specific wavelengths may modulate antioxidant activity and the synthesis of bioactive compounds [7,16]. Such spectral effects are increasingly relevant for medicinal and aromatic plants, where secondary metabolites determine both therapeutic efficacy and commercial value. Medicinal species like *Salvia officinalis* L. and *Cannabis sativa* L. are particularly suitable models for exploring light-driven modulation of biochemical and physiological traits. In order to improve yield and bioactive compound content, understanding the spectral regulation of physiological parameters in these species is crucial for sustainable indoor cultivation. Tailored light spectra can enhance resource-use efficiency, reduce energy consumption, and minimize the need for chemical inputs by promoting healthier, more resilient plants. Integrating knowledge of spectral effects into indoor practices can thus contribute to more environmentally sustainable and economically viable production systems for medicinal and aromatic plants. In this context, understanding how specific LED spectra affect physiological and biochemical traits is essential for developing efficient cultivation strategies. In this context, the present study aimed to disentangle the effects of LED spectral composition, with particular emphasis on green light deprivation and modulation of the red-to-blue (R:B) ratio at constant photon flux density, on plant morphology, mineral nutrient accumulation, and photosynthetic pigment biosynthesis in *Salvia officinalis* L. and *Cannabis sativa* L. grown under controlled conditions. We hypothesized that targeted spectral manipulation would elicit species-specific physiological and biochemical responses, reflecting different strategies of light perception and resource allocation, and thereby providing insights for the optimization of LED light recipes in indoor cultivation of medicinal plants. Therefore, investigating plant responses under green-light deprivation may help clarify whether the absence of this spectral region enhances or modifies the physiological and morphogenetic effects driven by red and blue wavelengths in controlled-environment cultivation systems.

## 2. Results

### 2.1. Salvia Officinalis

Table 1 reports the effects of light treatments on the fresh weight, leaf number, and plant height of *Salvia officinalis* L. plants. The first two parameters did not show a significant response to the different light conditions. The average fresh weight was 6.54 g/plant, while the average number of leaves was 5.5 per plant. Regarding plant height, the R:B_2.7_ treatment resulted in a 36% increase in height compared to the other treatments (*p* < 0.001).

The aluminum content did not vary significantly across treatments, averaging 3.17 mg/kg of fresh weight (FW). Boron levels increased significantly (*p* < 0.001), with R:B_1.6-NOGREEN_ showing the highest accumulation, followed by R:B_2.7_ and control, as reported in Table 2.

Iron concentrations were significantly higher in the R:B_1.6-NOGREEN_ treatment compared with the control (R:B_1.6_), showing increases of approximately 15%. Higher manganese values were found in the R:B_1.6-NOGREEN_ and R:B_2.7_ treatments, with a mean content of 3.77 mg/kg of FW. This value was 22% greater than that in the R:B_1.6_ treatment, as reported in Table 2.

Sodium content in *Salvia* was highest in plants grown under the R:B_1.6_ light treatment, followed by those under the R:B_2.7_ and R:B_1.6-NOGREEN_ treatments. In contrast, the contents of zinc (mean 2.74 mg/kg FW), calcium (mean 2.48 g/kg of FW), potassium (mean 7.98 g/kg FW), and magnesium (mean 1.56 g/kg of FW) showed no significant differences among treatments. Nitrogen content, however, was significantly enhanced by the spectral lighting. Compared with the control, the R:B_2.7_ treatment showed an increase of approximately 13%, whereas the R:B_1.6-NOGREEN_ treatment resulted in an increase of about 18% (*p* < 0.001). No significant differences were observed among treatments for chlorophyll a, chlorophyll b, and total chlorophyll content (on average 781–594 and 1375 µg/g of FW, respectively), as reported in Table 3. In contrast, a 22% increase in carotenoid content was observed in sage plants grown under the R:B_2.7_ treatment compared to those under the R:B_1.6_ and R:B_1.6-NOGREEN_ light treatments (112 µg/g of FW, on average).

As reported in Table 3, chloride content was not significantly affected by the treatments, with an average content of 3262 mg/kg of DW. Similarly, NO_3_ content did not differ significantly among treatments (*p* = 0.060), although a slight decreasing trend was observed. Relative to R:B_1.6_, nitrate content decreased by 3.4% in R:B_1.6-NOGREEN_ and by 8.8% in R:B_2.7_.

No significant differences were observed among treatments for phosphate (PO_4_) and sulfate (SO_4_) concentrations.

### 2.2. Cannabis sativa

Table 1 reports the effects of light treatments on the fresh weight, leaf number, and plant height of *C. sativa* plants. Fresh weight and plant height were not significantly affected by the light treatments, with mean values of 2.42 g per plant and 23.2 cm, respectively.

The number of leaves per plant was strongly affected by the light treatment, with the R:B_2.7_ condition producing twice as many leaves as the R:B_1.6_ and R: B_1.6-NOGREEN_ treatments (*p* < 0.001). The mineral composition of hemp demonstrated significant treatment effects across multiple elements, as reported in Table 2. Aluminum content increased significantly under R:B_2.7_ (+36%) compared to R:B_1.6_. Boron levels were significantly higher under both R:B_1.6-NOGREEN_ and R:B_2.7_, averaging 8.17 mg/kg FW, which is a 23% increase over R:B_1.6_. Regarding manganese content, the R:B_1.6-NOGREEN_ treatment resulted in a 36% increase compared to the R:B_1.6_ and R:B_2.7_ treatments (26.3 mg/kg of FW, on average).

Sodium content showed a significant increase (*p* < 0.01) under the R:B_2.7_ treatment, while the lowest value was recorded in R:B_1.6_. Light treatments did not alter the potassium content in hemp, with an average value of 9.85 g/kg of FW. Zinc, calcium, and magnesium levels were higher in the R:B_1.6-NOGREEN_ treatment, showing increases of 28%, 15%, and 16%, respectively, compared to plants grown under R:B_1.6_. Significant minor variations were also found in total nitrogen content. The R:B_1.6-NOGREEN_ treatment showed 7.5% more nitrogen compared to the R:B_1.6_ and R:B_2.7_ treatments (0.93 g/100 g of FW, on average).

The main photosynthetic pigments, chlorophyll a, chlorophyll b, and carotenoids, showed a similar variation trend, as reported in Table 3. The highest values were systematically found in the R:B_1.6-NOGREEN_ treatment compared to R:B_1.6_ and R:B_2.7_. Relative to the latter two, the “no green” treatment resulted in an increase of 12% in chlorophyll a, 9% in chlorophyll b, 11% in total chlorophyll, and 13% in total carotenoids.

As shown in Table 3, chloride content was highest in the R:B_1.6_ treatment, followed by R:B_1.6-NOGREEN_ and R:B_2.7_ (1997 mg/kg of DW). In contrast, NO_3_ concentration was not significantly affected by the treatments (*p* = 0.068); however, a decreasing trend was observed. Compared with R:B_1.6_, NO_3_ content declined by 16% under R:B_1.6-NOGREEN_ and by 19% under R:B_2.7_, with a mean concentration of 12,403 mg/kg of DW. No significant differences were observed among treatments for PO_4_ or SO_4_ concentrations. Their mean values were 2326 and 3800 mg/kg DW, respectively.

## 3. Discussion

This study investigated the influence of LED light spectral composition, specifically green light deprivation and different red-to-blue (R:B) ratios, on morphological development, mineral uptake, and pigment biosynthesis in hemp (*Cannabis sativa* L.) and sage (*Salvia officinalis* L). The observed responses, often species-specific, reflect complex interactions among photoreceptor-mediated signaling, hormonal regulation, and key metabolic pathways involved in plant growth and development. The tested lighting treatments did not result in significant differences in yield, expressed as fresh weight per plant, in either species. Although several studies have reported that the inclusion of green light can, in some cases, enhance biomass by partially replacing red and blue components at equal total light intensity [17,18], such effects are strongly species- and cultivar-dependent [19,20,21]. For instance, some tomato and lettuce cultivars, as well as basil, showed biomass increases, whereas no significant effects were found in other cultivars of cucumber, tomato, and lettuce [21].

In the present study, variation in the R:B ratio did not induce significant differences in total biomass under the adopted experimental conditions. Piovene et al. [22] reported that increasing the R:B ratio from 0.6 to 5.5 resulted in a linear reduction in biomass in basil, whereas in the same study, an R:B ratio of 1.5 led to a significant increase in leaf fresh weight in strawberry. It is plausible that longer growth periods could allow more pronounced differences to emerge, given that in the present study, plants were harvested after one month of treatment, which is a relatively short duration for long-cycle species such as salvia and hemp. In hemp, an increase in the R:B ratio promoted leaf production, likely through enhanced apical meristem activity and an accelerated leaf appearance rate rather than through increased nodal density. In sage, the increase in plant height observed at higher R:B ratios can be attributed to enhanced phytochrome-mediated internode elongation and reduced cryptochrome inhibition, a photomorphogenic response well documented in aromatic species of the genus Salvia. Lee et al. [23] reported that in *Salvia plebeia*, an R:B ratio of 2.3 improved several growth traits, including leaf area, leaf length, biomass, and photosynthetic rate. In contrast, in the present study, *Salvia officinalis* exhibited a distinct response to an R:B ratio of 2.7, characterized by marked stem elongation (32–40% increase in plant height) without concomitant changes in leaf number or biomass accumulation. Similar responses have been reported in tomato, cucumber, and pepper [24] and may be attributed to a reduced proportion of blue light, which is required to promote compact growth and to minimize shade-avoidance responses such as excessive stem elongation [25,26,27].

The lighting treatments induced statistically significant variations in the content of the major mineral elements analyzed. Compared with the R:B_1.6_ treatment, both green light deprivation and increasing the R:B ratio generally promoted higher mineral accumulation. Consistent with these results, basil grown indoors under R:B ratios ranging from 0.5 to 4.0 exhibited a progressive rise in the levels of N, P, K, Ca, Mg, and Fe as the R:B ratio became higher [28]. Likewise, the R:B_1.6_ treatment without green light generally resulted in higher macro- and micronutrient contents, in agreement with studies reporting that increasing the proportion of blue light at equal light intensity can enhance mineral accumulation, as observed in pakchoi and mustard microgreens [29]. These increases may be attributed to the ability of blue light to modulate stomatal opening and membrane transporter activity, thereby enhancing nutrient uptake and translocation, as also reported in broccoli microgreens [30]. Blue light exposure is additionally associated with changes in Ca^2+^, K^+^, and H^+^ fluxes in guard cells, leading to modifications in pH conditions and ion transport activity [31]. In addition, the deprivation of green light may have enhanced the effect of blue light, since both wavelengths compete for the same photoreceptors [6,7], thereby promoting the downstream activation of the physiological processes triggered by this light stimulus.

For both species and under all treatments, the concentrations of Ba, Cd, Cr, Co, and Pb were below the limit of detection, highlighting the nutritional safety of the soilless cultivation system. Although spectral variations are known to significantly influence the content of photosynthetic pigments, the changes observed in the present study were relatively limited. Nevertheless, the significant differences in chlorophyll a, chlorophyll b, and total chlorophyll content observed in hemp under the R:B_1.6_ and R:B_2.7_ treatments compared with those including green light suggest that the green component may attenuate the specific stimulatory effects of red and blue wavelengths, as reported by Schmalstig et al. [32], in *Arabidopsis thaliana* and *Landoltia punctata*. The increases in carotenoid content observed in both hemp and salvia could be associated with moderate photostress, a known trigger of carotenoid accumulation under suboptimal light environments. This effect may be related to the specific spectral balance to which the plants were exposed. For example, green-light deprivation (R:B_1.6 NOGREEN_) in hemp or a high proportion of red light (R:B_2.7_) in sage may have created a slight imbalance in the excitation of the two photosystems or in developmental signaling. Such a condition could have triggered acclimation responses, including an increase in photoprotective pigments such as carotenoids. Furthermore, the increase in carotenoids may also result from an interaction between blue and green light, since, as reported by several authors, both of these light signals compete for the same cryptochromes (CRYs) [6,7].

It is well established that light quality can stimulate carotenoid biosynthesis as part of the plant’s photoprotective response, as reported in lettuce [33]. With respect to nitrate (NO_3_) content, a key parameter for the nutritional quality of leafy vegetables and for compliance with current regulations, mean values were 2469 ± 269 mg/kg of FW in hemp and 5945 ± 207 mg/kg of FW in salvia. These concentrations were below the maximum limit of 7000 mg/kg of FW established by current legislation. The observed trend toward NO_3_ reduction, although not statistically significant (*p* = 0.068 in hemp and *p* = 0.060 in sage), is consistent with previous studies. Several studies indicate that different R:B ratios can reduce the concentration of this antinutrient [34], whose intake is associated with the formation of potentially harmful nitrosamines. It is plausible that green light deprivation results in a higher effective photon flux of red and blue light, whose combined action appears particularly effective in limiting NO_3_ accumulation, as also confirmed by [35].

## 4. Materials and Methods

### 4.1. Experimental Design and Plant Material

The experiment was conducted in a walk-in growth chamber located at the Institute of Sciences of Food Production (ISPA-CNR) in Bari (BA), with internal dimensions of 260 cm × 284 cm × 253 cm (length, width, and height, respectively). A randomized complete block experimental design with three replicates per treatment was adopted. For each species, a total of 90 plants were cultivated, corresponding to 30 plants per treatment. Environmental conditions were maintained at 22 °C and 65% relative humidity throughout the experiment, with a photoperiod of 16 h of light followed by 8 h of darkness, providing consistent daylength conditions for both species.

*Salvia officinalis* L. seeds (Seeds Select Pro) were initially sown in 160-hole plug trays, filled with peat, and grown for 40 days. During the first three days, lights were kept off to allow the seeds to germinate in the dark. On day 4, the seedlings were exposed to artificial lighting conditions comprising 14 h of light and 10 h of darkness. The light period included 1 h of dawn, with a photosynthetic photon flux density (PPFD) of 125 µmol/s/m^2^, followed by 12 h of full light at a PPFD of 232 µmol/s/m^2^, and 1 h of dusk with a PPFD of 125 µmol/s/m^2^. Seedlings were subsequently transplanted into 30 L grow bags filled with a 50% *v*/*v* mixture of coco fiber and perlite (Agripan, Perlite Italiana, Corsico, Italy), with each bag accommodating ten plants. Three grow bags per treatment were used.

*Cannabis sativa* L. cultivar ‘Nashinoide’ seeds (CREA-DC-I) were sown in 160-hole plug trays, filled with peat, using the same environmental conditions used for *S. officinalis* seedlings, for 20 days before transplanting them into 0.5 L pots, containing a 3:1 *v*/*v* mixture of peat and perlite. In both trial experiments, the plants were fertigated with full-strength Hoagland nutrient solution (NS), with the following composition: 224 mg/L N, 235 mg/L K, 160 mg/L Ca, 62 mg/L P, 32 mg/L S, 24 mg/L Mg, 0.270 mg/L B, 1.12 mg/L Fe, 0.110 mg/L Mn, 0.131 mg/L Zn, 0.032 mg/L Cu, and 0.050 mg/L Mo [36].

At the time of transplanting, the light treatments summarized in Table 4 were initiated. All light intensities and spectral distributions were measured and calibrated at the canopy level using quantum sensors to ensure uniformity across treatments and to minimize spatial variation in light exposure throughout the cultivation period.

### 4.2. Data Collection and Determination of Chlorophylls and Carotenoid Content

Both *S. officinalis* and *C. Nashinoide* plants were harvested one month after transplanting. Afterwards, morphological parameters including plant height, fresh weight, and number of leaves were recorded for both species. Chlorophylls and total carotenoid content were extracted by using an acetone solution and determined spectrophotometrically, as reported by Montesano et al. [37].

### 4.3. Analysis of Total Nitrogen and Inorganic Elements

The Cl, NO_3_, PO_4_ and SO_4_ ions were determined by ion exchange chromatography technique (IC-Dionex DX120, Dionex Corporation, Sunnyvale, CA, USA) with a conductivity detector, as reported by D’Imperio et al. [38]. For Al, K, Ca, P, Fe, Mg, Na, B, Cd, Co, Cr, Cu, Li, Mn, Mo, Pb, and Zn determinations, *S. officinalis* and *C. sativa* freeze-dried samples were digested in a closed-vessel microwave digestion system (MARS 6, CEM Corporation, Matthews, NC, USA) with 10 mL of HNO_3_ (Pure grade, Carlo Erba) and analyzed by using inductively coupled plasma–optical emission spectrometry, as reported by D’Imperio et al. [39].

The total nitrogen content was determined using a CN 802 analyzer (VELP, Milan, Italy). Briefly, 100 mg of freeze-dried sample were placed into tin foil (VELP Scientifica Srl, Usmate Velate, Italy) and deposited into the autosampler. Five analytical standards for nitrogen detection were prepared using increasing concentrations of EDTA (VELP Scientifica Srl, Usmate Velate, Italy), ranging from 10 to 100 mg of powder. A blank made with empty tin foil was also adopted.

To confirm the accuracy of the measurements of total N, NO_3_, Al, K, Ca, P, Fe, Mg, Na, B, Cd, Co, Cr, Cu, Mn, Mo, Li, Pb, and Zn, certified reference vegetable material (CRM, NIST tomato leaf 1535a) was analyzed using the same procedure adopted for freeze-dried samples.

### 4.4. Statistical Analysis

Effects of different treatments were tested using analysis of variance (ANOVA), followed by means separation with Tukey’s HSD test at *p* ≤ 0.05. The statistical software STATISTICA 10.0 (StatSoft, Tulsa, OK, USA) was used for the analysis. The percentage coefficient of variation (CV %) was determined according to the method described by Gomez and Gomez [40].

## 5. Conclusions

This study shows that targeted manipulation of LED spectral composition through green light deprivation and modulation of the red-to-blue (R:B) ratios at constant photon flux density induces clear and species-specific responses in *Salvia officinalis* and *Cannabis sativa*. While total biomass was not significantly affected, spectral changes markedly influenced specific morphological traits, such as stem elongation in *S. officinalis* and leaf production in *C. sativa*, mineral nutrient accumulation, and pigment biosynthesis. A higher R:B ratio promoted elongation in *S. officinalis* and increased leaf production in *C. sativa*, confirming distinct developmental strategies driven by light signaling. Green light deprivation and red–blue enrichment generally increased mineral accumulation and total nitrogen, while pigment adjustments were more pronounced in hemp than in sage.

Overall, the results highlight the importance of species-specific optimization of light spectra to improve physiological performance and nutritional quality in controlled-environment cultivation of *Salvia officinalis* and *Cannabis sativa*. These findings provide a scientific basis for the development of tailored LED light recipes aimed at optimizing plant architecture, mineral composition, and biochemical quality in sustainable indoor cultivation systems.

## Figures and Tables

**Table 1:** Biometric parameters of *Salvia officinalis* L. and Hemp (*Cannabis Sativa* L. cv. Nashinoide) cultivated with different LED light treatments differing in red:blue ratios (R:B) and green light deprivation (R:B_1.6-NOGREEN_).

Treatments	Fresh Weight	Number of Leaves	Plant Height
	g/Plant	n/Plant	cm/Plant
*Salvia officinalis* L.			
R:B_1.6_	5.83 ± 0.77	5.1 ± 0.94	16.0 ± 1.00 b
R:B_1.6-NOGREEN_	7.31 ± 0.57	4.9 ± 1.19	16.9 ± 1.02 b
R:B_2.7_	6.48 ± 1.2	6.4 ± 1.23	22.4 ± 0.83 a
Significance	ns	ns	***
CV%	28.4	24.4	7.3
*Cannabis Sativa* L. cv. Nashinoide			
R:B_1.6_	2.77 ± 0.26	9.5 ± 0.13 b	24.4 ± 1.06
R:B_1.6-NOGREEN_	2.31 ± 0.03	8.4 ± 0.23 b	22.0 ± 1.07
R:B_2.7_	2.19 ± 0.06	19.2 ± 0.81 a	23.1 ± 0.49
Significance	ns	***	ns
CV%	10.1	6.6	6.5

Data are expressed as mean of three independent elementary units. Significance: ns, not significant; *** *p* < 0.001. Different letters indicate that the mean values are significantly different, according to the LSD test (α = 0.05). CV% coefficient of variation percentage.

**Table 2:** Mineral composition and total nitrogen content of *Salvia officinalis* L. and Hemp (*Cannabis Sativa* cv. Nashinoide) cultivated with different LED light treatments differing in red:blue ratios (R:B) and green light deprivation (R:B_1.6-NOGREEN_).

Treatments	Al	B	Fe	Mn	Na	Zn	Ca	K	Mg	N
	mg/kg of FW						g/kg of FW			g/100 g of FW
*Salvia officinalis* L.										
R:B _1.6_	3.54 ± 0.13	6.19 ± 0.1 b	10.5 ± 0.15 b	3.10 ± 0.0 b	184 ± 2.7 a	3.01 ± 0.19	2.44 ± 0.52	7.77 ± 0.11	1.52 ± 0.00	0.60 ± 0.01 c
R:B_1.6-NOGREEN_	2.94 ± 0.42	6.93 ± 0.1 a	12.1 ± 0.29 a	3.82 ± 0.0 a	91 ± 1.9 c	2.54 ± 0.0	2.88 ± 0.41	8.10 ± 0.29	1.71 ± 0.1	0.71 ± 0.01 a
R:B_2.7_	3.03 ± 0.21	6.58 ± 0.0 ab	11.2 ± 0.1 ab	3.72 ± 0.0 a	113 ± 0.9 b	2.67 ± 0.0	2.12 ± 0.18	8.07 ± 0.20	1.48 ± 0.05	0.68 ± 0.02 b
Significance	ns	***	**	***	***	ns	ns	ns	ns	***
CV%	10.7	3.2	5.5	7.3	25.3	8.2	22.8	3.9	7.5	5.3
*Cannabis Sativa* cv. Nashinoide										
R:B_1.6_	2.69 ± 0.22 b	6.64 ± 0.53 b	22.7 ± 7.4	23.6 ± 2.53 b	9.24 ± 2.08 b	3.60 ± 0.42 b	10.5 ± 1.0 b	8.60 ± 0.87	1.96 ± 0.20 b	0.92 ± 0.03 b
R:B_1.6-NOGREEN_	3.29 ± 0.28 ab	8.32 ± 0.06 a	18.3 ± 0.39	35.8 ± 0.06 a	12.3 ± 0.20 ab	4.60 ± 0.06 a	14.3 ± 0.35 a	11.0 ± 0.18	2.66 ± 0.06 a	1.00 ± 0.08 a
R:B_2.7_	3.67 ± 0.42 a	8.03 ± 0.18 a	16.8 ± 0.54	29.0 ± 0.92 b	17.3 ± 0.77 a	3.94 ± 0.17 ab	12.1 ± 0.20 ab	9.95 ± 0.11	2.27 ± 0.03 ab	0.94 ± 0.05 b
Significance	*	*	ns	***	**	*	*	ns	*	**
CV%	8.5	5.9	18.1	8.9	16.7	8.6	8.4	7.4	7.7	2.6

Data are expressed as mean of three independent elementary units. Significance: ns, not significant; * *p* < 0.05, ** *p* < 0.01; *** *p* < 0.001. Different letters indicate that the mean values are significantly different, according to the LSD test (α = 0.05). FW: fresh weight. CV% coefficient of variation percentage.

**Table 3:** Principal pigment and anion content of *Salvia officinalis* L. and Hemp (*Cannabis Sativa* cv. Nashinoide) cultivated with different LED light treatments differing in red:blue ratios (R:B) and green light deprivation (R:B_1.6-NOGREEN_).

Treatments	CHLa	CHLb	CHLtot	Carotenoids	Cl	NO_3_	PO_4_	SO_4_
	µg/g of FW				mg/kg of DW			
*Salvia officinalis* L.								
R:B_1.6_	755 ± 40.2	590 ± 26.3	1346 ± 65.6	110 ± 0.49 b	2907 ± 55	46,133 ± 1007	10,573 ± 221	2713 ± 103
R:B_1.6-NOGREEN_	761 ± 21.2	580 ± 9.9	1341 ± 30.9	114 ± 4.53 b	3387 ± 81	44,553 ± 572	11,260 ± 1071	2053 ± 297
R:B_2.7_	827 ± 16	612 ± 25.5	1439 ± 41.7	137 ± 1.12 a	3494 ± 322	42,059 ± 591	10,955 ± 331	2404 ± 191
Significance	ns	ns	ns	***	ns	ns (*p* = 0.060)	ns	ns
CV%	5.2	4.7	4.6	9.2	9.7	5.1	8.9	13.9
*Cannabis Sativa* cv. Nashinoide								
R:B_1.6_	1323 ± 13.1 b	766 ± 18.42 b	2089 ± 30.9 b	341 ± 1.40 b	2693 ± 175 a	14,993 ± 375	3087 ± 1218	3493 ± 565
R:B_1.6-NOGREEN_	1492 ± 4.4 a	845 ± 4.25 a	2337 ± 5.26 a	381 ± 2.48 a	1933 ± 128 b	12,627 ± 627	1807 ± 320	3593 ± 195
R:B_2.7_	1331 ± 15.07 b	783 ± 15.95 b	2114 ± 30.39 b	335 ± 1.35 b	2061 ± 42 b	12,180 ± 621	2085 ± 39	4315 ± 282
Significance	***	**	***	***	*	ns (*p* = 0.068)	ns	ns
CV%	4.5	3.8	4.1	4.6	12	9.9	47.4	10.1

Data are expressed as mean of three independent elementary units. Significance: ns, not significant; * *p* < 0.05; ** *p* < 0.01; *** *p* < 0.001. Different letters indicate that the mean values are significantly different, according to the LSD test (α = 0.05). FW: fresh weight; DW: dry weight. CV% coefficient of variation percentage.

**Table 4:** Description of the light treatments applied in the two agronomic trials.

Treatments	Blue	Green	Red	PPFD	DLI	Ratio Red:Blue
	(%)			µmol m^−2^ s^−1^	mol⋅m^−2^⋅d^−1^	
R:B_1.6_	22	43	35	232	13	1.6
R:B_1.6 NOGREEN_	38	0	62	232	13	1.6
R:B_2.7_	16	41	43	232	13	2.7

PPFD: Photosynthetic Photon Flux Density; DLI: Daily light integral. Spectral photon flux distributions for the lighting treatments. Green light-emitting diodes (LEDspeak wavelength ranged from 520 to 540 nm), red light-emitting diodes (LEDspeak wavelength 655 nm and blue light-emitting diodes (LEDspeak wavelength 452 nm). DLI calculated by considering, in addition to the 12 h of light at 232 µmol·s^−1^·m^−2^, the sunrise (1 h) and sunset (1 h) phases with an intensity of 125 µmol·s^−1^·m^−2^. They use the following calculation: (PPFD × seconds per day × hours of light)/1,000,000. DLI_tot_ = 10.02 + 0.45 + 0.45 = 10.92 mol⋅m^−2^⋅d^−1^.

## Data Availability

Data are contained within the article.
